# Bioinformatics in Italy: BITS2011, the Eighth Annual Meeting of the Italian Society of Bioinformatics

**DOI:** 10.1186/1471-2105-13-S4-I1

**Published:** 2012-03-28

**Authors:** Paolo Romano, Manuela Helmer-Citterich

**Affiliations:** 1 Bioinformatics, IRCCS AOU San Martino - IST National Cancer Research Institute, Genoa, I-16132, Italy; 2 Department of Biology, University of Rome "Tor Vergata", Rome, I-00133, Italy

## Abstract

The BITS2011 meeting, held in Pisa on June 20-22, 2011, brought together more than 120 Italian researchers working in the field of Bioinformatics, as well as students in Bioinformatics, Computational Biology, Biology, Computer Sciences, and Engineering, representing a landscape of Italian bioinformatics research.

This preface provides a brief overview of the meeting and introduces the peer-reviewed manuscripts that were accepted for publication in this Supplement.

## Preface

### The Italian Society of Bioinformatics

The Italian Society of Bioinformatics (BITS) [1] was founded in 2003 by a small group of Italian scientists, engaged in various disciplines ranging from physics to informatics and molecular biology. Since then, the number of involved researchers was continuously increased year after year. The Society has now about 230 members and aims at overcoming 250 in 2012.

The main aim of the Society, which is a Regional group of the International Society for Computational Biology (ISCB), is the fostering of Bioinformatics in Italy. Its activities include the organization of an annual scientific meeting, the maintenance of a web site and of a mailing list for the distribution of news of interest for the involved community of researchers, the coordination of educational initiatives in Italy, from bachelor to PhD degrees, the coordination of research activities among members, and the improvement of the participation of Italian researchers, both senior and junior, to international events and projects of relevance.

### The Eighth BITS Meeting: BITS 2011

The Eighth Annual BITS Meeting, BITS 2011, was held in Pisa at the Research Campus (Area della Ricerca) of the National Research Council (CNR), on June 20-22, 2011. The meeting was organized by Marco Pellegrini of the CNR Institute for Informatics and Telematics and by Roberto Marangoni of the Computer Science Department of the University of Pisa.

Over 120 scientists actively working in bioinformatics and related fields or strongly interested in its development met and discussed their work, state of the art and future perspectives. A total of 98 abstracts were accepted: 23 of them were selected for oral presentation by the Scientific Committee after a well established peer-review procedure based on three reviews and scores per paper. The remaining 75 were presented in the poster sessions.

Four keynote talks were given by distinguished scientists. Marie-France Sagot, INRIA-Lyon, France, gave a talk on "Towards an Algorithmic and Mathematical Exploration of Symbiosis". "How not to Become a Systems Biologist" was the title of the "Giuliano Preparata" Lecture given by Arthur Lesk, Penn State University, USA. Bud Mishra, New York University, USA, gave a provocative talk on "Why we Keep Assembling...". "Using the T-Coffee Multiple Sequence Aligner in the High Throughput Era" was the title of the keynote given by Cedric Notredame, Center for Genomic Regulation (CRG), Barcelona, Spain. The last talk was co-sponsored by the Italian Network for Bioinformatics Oncology (RNBIO).

The conference was organized into thematic sessions that reflected the following conference topics: Genomics, Molecular Evolution and Comparative Genomics, Protein structure and function, Proteomics, Transcriptomics, Metagenomics, Systems Biology, Biological Databases, Biobanks, Algorithms for Bioinformatics, Biophysics, and Synthetic Biology.

An "industrial track" was also organized under the form of a round table focused at opening a dialogue between the industries and the academic world on bioinformatics. The round table was participated by deputies of University of Pisa, CNR, industrial accelerators programs, and computing and/or life sciences companies in Tuscany. It ended with the decision to open a permanent table of discussion on the advancement of bioinformatics in the Innovation Poles in Tuscany.

The web site [2] of the meeting includes the video recording of the vast majority of oral presentations.

Two tutorial lectures were also given on the last day of the meeting at the Computer Science Department of the University of Pisa. Giorgio Valentini, from the University of Milan (Italy), gave a tutorial on "Machine learning methods for gene function prediction" and Andrea Bracciali, from the University of Stirling (Scotland, UK), gave a lecture on "Formal Models in Systems Biology".

### The selection of papers for this Supplement

Shortly after the conference, 42 papers were submitted for publication in this BMC Bioinformatics Supplement. An Editorial Board was formed, including all members of BITS Programme Committee. Associated Editors are listed here:

• Rita Casadio, University of Bologna, Bologna, Italy

• Gianni Cesareni, University of Rome "Tor Vergata", Rome, Italy

• Francesca Ciccarelli, European Oncology Institute, Milan, Italy

• Domenica D'Elia, CNR Institute for Biomedical Technologies, Bari, Italy

• Diego Di Bernardo, Telethon Institute of Genetics and Medicine, Naples, Italy

• Angelo Facchiano, CNR Institute of Food Sciences, Avellino, Italy

• Manuela Helmer Citterich, University of Rome "Tor Vergata", Rome, Italy

• Sabino Liuni, CNR Institute for Biomedical Technologies, Bari, Italy

• Roberto Marangoni, University of Pisa, Pisa, Italy

• Marco Pellegrini, CNR Institute for Informatics and Telematics, Pisa, Italy

• Graziano Pesole, University of Bari, Bari, Italy

• Paolo Romano, IRCCS San Martino IST, Genova, Italy

• Giorgio Valle, University of Padova, Padova, Italy

A stringent reviewing procedure was then adopted. Associate Editors handled the process according to their recognized knowledge in specific meeting topics. At least three referees, of a high reputation at an international level, were selected for each submission. Overall, 67 referees from 20 different countries were involved in the selection of papers. We opted for a two step peer review procedure, offering authors the possibility to submit a new version of their paper, revised according to the referees' comments.

At the end of this process, 22 papers were accepted and are now included in this Supplement. They cover different aspects of theoretical and applied Bioinformatics. For sake of readability, they are presented in this Supplement grouped by topic.

#### Algorithms for Bioinformatics

In the context of "Algorithms for Bioinformatics", we present three contributions. "Accurate multiple sequence alignment of transmembrane proteins with PSI-Coffee" from Chang *et al *[3] reports a new strategy to align alpha-helical trans-membrane proteins based on homology extension and PSI-Coffee that shows a significant improvement over the most accurate methods currently available. "PyMod: sequence similarity searches, multiple sequence-structure alignments, and homology modeling within PyMOL" by Bramucci *et al*. [4] presents a simple interface between PyMOL and several bioinformatics tools showing how complex processes, including homology modeling and sequence/structure analysis, can be greatly simplified when integrated into the PyMOL framework. "Tandem repeats discovery service (TReaDS) applied to finding novel cis-acting factors in repeat expansion diseases" by Pellegrini *et al*. [5] presents a meta search engine that queries various tandem repeat resources and merges their outcome, thus producing a unified, comparative view of results, and its application for the analysis of sequences associated with repeat expansion diseases.

#### Biological Databases and Biobanks

In the context of "Biological Databases and Biobanks", this Supplement includes the following four papers. "GIDL: a rule based expert system for GenBank Intelligent Data Loading into the Molecular Biodiversity Database" by Pannarale *et al *[6] presents a system that is meant to populate the Molecular Biodiversity Database with information extracted from GenBank by means of a semantic-based tool, the Intelligent Data Loader, that is able to manage entities from the Sequence Ontology and the Chado relational schema for Generic Model Organism Databases.

"An ICT infrastructure to integrate clinical and molecular data in oncology research" by Segagni *et al *[7] presents the extensions introduced to the bioinformatic platform i2b2, a system designed to integrate clinical and research data, providing synchronization with a biobank database and integration of coded information and clinical results from unstructured medical records by means of a NLP module.

"OREMPdb: a semantic dictionary of computational pathway models" by Umeton *et al *[8] presents an application of OREMP (Ontology Reasoning Engine for Molecular Pathways) to the curated branch of BioModels, that includes 326 models for thousands of reactions and species involved.

"Towards Linked Open Gene Mutations Data" by Zappa *et al*. [9] presents a prototype implementation of the IARC TP53 Mutation database as Linked Open Data (LOD) that allows to semantically integrate mutation data with information from public molecular biology databases whose content is already available through the LOD and can also serve as a starting point for the development of an ontology on human variation data.

#### Biophysics and Synthetic Biology

In the context of "Biophysics and Synthetic Biology", the paper "Fine-tuning anti-tumor immunotherapies via stochastic simulations" by Caravagna *et al *[10] uses simulation tools to optimize the efficiency of immunotherapy in cancer treatments. "Characterization of the emergent properties of a synthetic quasi-cellular system" by Lazzerini-Ospri *et al *[11] presents a theoretical forecast of an experiment that will discriminate between Poisson and non-Poisson distribution of solutes in small-size vesicles. Finally, "Stochastic simulations of minimal cells: the Ribocell model" by Mavelli [12] offers a description of a minimal cell based on RNAs with enzymatic activity, and "Characterization of an inducible promoter in different DNA copy number conditions" by Zucca *et al *[13] copes with the unpredictability of the genetic circuitry when assembled and incorporated in living cells, which is one of the main problems in synthetic biology, by trying to disclose the linearity working boundaries when dealing with some of the most relevant biological phenomena.

#### Genomics

For the "Genomics" topic, three papers were selected. In the first one, "In-silico and in-vivo analyses of EST databases unveil conserved miRNAs from *Carthamus tinctorius *and *Cynara cardunculus*" [14], Catalano and co-authors identified a number of miRNA and targets conserved in artichoke and safflower. Four highly significant miRNAs were experimentally validated in artichoke leaves. In the second paper, by Cornero *et al *[15], the authors present a classifier able to predict the expected prognosis in neuroblastoma patients. The classifier, based on an ensemble of gene signatures, determines patients' outcome with 94% accuracy. "Argot2: a large scale function prediction tool relying on semantic similarity of weighted Gene Ontology terms" by Falda *et al *[16] describes a web-based function prediction tool able to annotate nucleic or protein sequences also on genomic scale with GO terms weighted with the statistical significance of BLAST and HMMer search results.

#### Molecular Evolution and Comparative Genomics

For the "Molecular Evolution and Comparative Genomics" topic, "Primates and Mouse NumtS in the UCSC Genome Browser" from Simone *et al *[17] describes the annotation of NumtS sequences (mitochondrial sequences which have colonized the nuclear DNA) in the primates and mouse genomes.

#### Protein Structure and Function

The topic "Protein Structure and Function" had four works selected. Andrei *et al*., in "Intuitive representation of surface properties of biomolecules using BioBlender" [18], describe an interesting tool which is based on 3D animation and rendering software mixed with biochemical knowledge to represent protein surface properties. Features such as electrostatic potential or hydropathy can be simultaneously visualized in animated form. The second paper, by Bianchi *et al*. [19], describes a novel method for the identification of protein binding pockets, which outperforms other state-of-the-art methods when applied to unbound structures. The third paper, by Fogolari *et al*. [20], describes the BLUUES program for the analysis of the electrostatic properties of proteins based on generalized Born *radii*. A version of the software running on Linux is also made available for download as supplementary material. The last paper in this topic, by Vangone *et al *[21], reports a novel tool to measure and visualize the conservation of inter-residue contacts in multiple docking solutions; the CONS-COCOMAPS program can provide an immediate graphical view of the consensus of many different predictions, for a given complex, through an effective "consensus map".

#### System Biology

The "Systems Biology" topic is represented by the work of Fioravanti and co-authors [22], where state-charts, a modular, hierarchical and executable formal model widely used to represent software systems, is applied to the description of Gene Regulatory Networks (GRN). State-charts are shown to be able to simulate some interesting properties and temporal dynamics of GRN motifs, such as simple regulation, reciprocal regulation, feedback loop, feedforward loop and auto regulation.

#### Transcriptomics

Finally, the "Transcriptomics" topic has two papers selected. The work by Consiglio *et al *[23] describes a web tool for the analysis and visualization of gene chip human exon array data from disease experiments. This tool offers advanced statistical features on a user-friendly platform. The work by Sanavia *et al*. [24] describes a novel classifier for the analysis of prognostic/diagnostic data for diseases, based on the selection of features that are shown to be stable in different biological datasets.

### Next meeting

The next Annual meeting of the Italian Society of Bioinformatics will be held in Catania, May 2-4, 2012. Further information about BITS 2012 is available on its purpose web site [25], as well as on our web site [1].
